# Novel Ferrocene Derivatives Induce G0/G1 Cell Cycle Arrest and Apoptosis through the Mitochondrial Pathway in Human Hepatocellular Carcinoma

**DOI:** 10.3390/ijms22063097

**Published:** 2021-03-18

**Authors:** Jianrong Zheng, Liao Zeng, Mingqing Tang, Hongjun Lin, Chao Pi, Ruian Xu, Xiuling Cui

**Affiliations:** 1 Engineering Research Centre of Molecular Medicine, Ministry of Education, Fujian Key Laboratory of Molecular Medicine, Key Laboratory of Precision Medicine and Molecular Diagnosis of Fujian Universities, Xiamen Key Laboratory of Marine and Gene Drugs, School of Medicine, Huaqiao University, Xiamen 361021, China; 18014071027@stu.hqu.edu.cn (J.Z.); 18014071002@stu.hqu.edu.cn (L.Z.); 19014071011@stu.hqu.edu.cn (H.L.); ruianxu@hqu.edu.cn (R.X.); 2 Henan Key Laboratory of Chemical Biology and Organic Chemistry, Key Laboratory of Applied Chemistry of Henan Universities, Green Catalysis Center, College of Chemistry, Zhengzhou University, Zhengzhou 450052, China; pichao@zzu.edu.cn

**Keywords:** human hepatocellular carcinoma, apoptosis, cell cycle, reactive oxygen species, mitochondrial membrane permeabilization

## Abstract

In this study, detailed information on hepatocellular carcinoma (HCC) cells (HepG-2, SMMC-7721, and HuH-7) and normal human liver cell L02 treated by ferrocene derivatives (compounds **1**, **2** and **3**) is provided. The cell viability assay showed that compound **1** presented the most potent and selective anti-HCC activity. Further mechanism study indicated that the proliferation inhibition effect of compound **1** was associated with the cycle arrest at the G0/G1 phase and downregulation of cyclin D1/CDK4. Moreover, compound **1** could induce apoptosis in HCC cells by loss of mitochondrial membrane potential (ΔΨm), accumulation of reactive oxygen species (ROS), decrease in Bcl-2, increase in BAX and Bad, translocation of Cytochrome *c*, activation of Caspase-9, -3, and cleavage of PARP. These results indicated that compound **1** would be a promising candidate against HCC through G0/G1 cell cycle arrest-related proliferation inhibition and mitochondrial pathway-dependent apoptosis.

## 1. Introduction

Hepatocellular carcinoma (HCC) is the sixth most commonly diagnosed of malignant tumors worldwide [1], with approximately 841,000 new cases in 2018 [2]. So far, HCC remains difficult to treat, owing to the poor response and severe toxicity of currently available chemotherapeutic drugs [3]. Sorafenib, a broad-spectrum kinase inhibitor, has been the first-line chemotherapeutic drug that can provide modest benefits for patients with advanced HCC; however, this drug does have limited effects and inevitably causes toxic reactions [4]. Toxic reactions caused by sorafenib have become increasingly prominent in clinical treatment, particularly hand–foot syndrome [5]. Therefore, it is urgent to develop a novel agent with low toxicity and high efficacy for the treatment of HCC.

Ferrocene derivatives are becoming more and more popular in medicinal molecules due to their unique chemical structures, biological activities, low toxicity, and reversible redox behavior [6]. Some compounds were found to exhibit a variety of pharmacological properties, including antibacterial [7], antimalarial [8], antifungal [9], antiviral [10], and anticancer activities [11]. Fortunately, we have successfully developed novel protocols to build a series of ferrocene derivatives based on transition metal-catalyzed C-H functionalization [12], and have explored the bioactivities of ferrocenyl olefins [13].

Herein, we embarked on examining the anti-HCC activity of the ferrocene derivatives obtained in our group, especially their underlying mechanism. The most important and interesting observation was that the IC_50_ of compound **1** against HCC cells (~20 μM) was far smaller than that of L02 (~210 μM), which indicated its potent anti-HCC activity and good selectivity. The selectivity of compound **1** was more striking when compared to the IC_50_ of sorafenib against L02 (0.35 μM). Furthermore, the antiproliferative effect of compound **1** was associated with G0/G1 phase cell cycle arrest and the intrinsic apoptotic pathway, i.e., decrease in Bcl-2 protein, increase in BAX and Bad protein, accumulation of reactive oxygen species (ROS), loss of mitochondrial membrane potential (ΔΨm), translocation of Cytochrome *c,* activation of Caspase-3 and -9, and cleavage of poly ADP-ribose polymerase (PARP). Thus, these findings indicated that compound **1** might represent a promising anti-HCC agent.

## 2. Results

### 2.1. Compound ***1*** Was the Most Potent and Selective Compound

**Figure 1:**
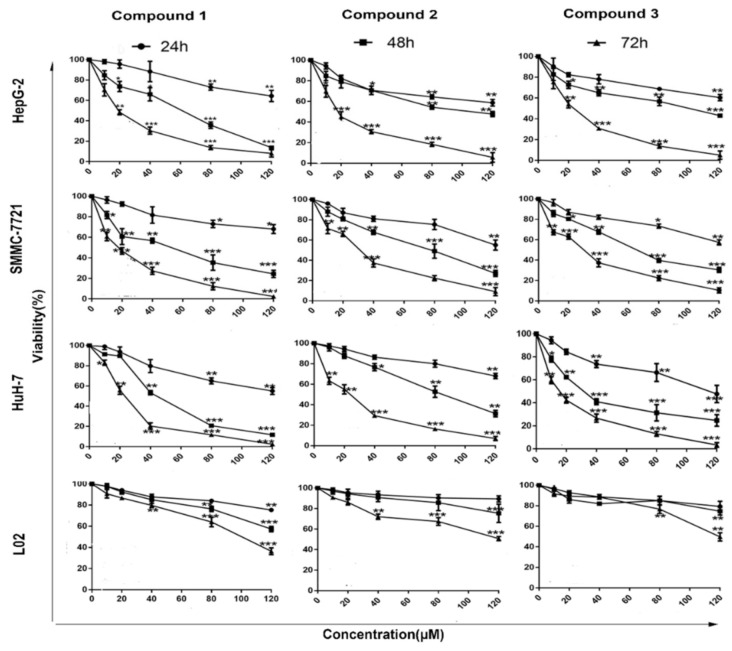
The viability of HCC cells and L02 cells treated with compounds **1**, **2**, and **3**. The viability of HCC cells and L02 cells treated by compounds **1**, **2**, and **3** (0, 20, 40, 60, 80 and 120 μM). Cell viability values are expressed as mean ± SD (*n* = 3). Significant differences from the control are indicated by * *p* < 0.05, ** *p* < 0.01, *** *p* < 0.001.

Poor selectivity mainly limits the clinical application of existing anticancer drugs [14]. We therefore evaluated the selectivity and anti-HCC potential of the compounds previously prepared in our group. The cell viability of three HCC cells (HepG-2, SMMC-7721 and HuH-7) and a normal human liver cell L02 was analyzed after being treated by compounds **1**, **2** and **3**, respectively. As shown in Figure 1, the proliferation of HCC cells was significantly inhibited by these compounds in a dose- and time-dependent manner. Interestingly, compound **1** presented the most potential selectivity, which was approximately 10-fold selectivity in inhibiting the growth of HepG-2 cells vs. L02 cells (40 μM, 72 h). The IC_50_ of sorafenib against L02 (0.35 μM) also confirmed compound **1** had a higher anti-HCC selectivity (Table S1). Therefore, compound **1** was chosen for the following molecular mechanism study.

### 2.2. Cell Cycle Arrest at G0/G1 Phase Treated with Compound ***1***

**Figure 2:**
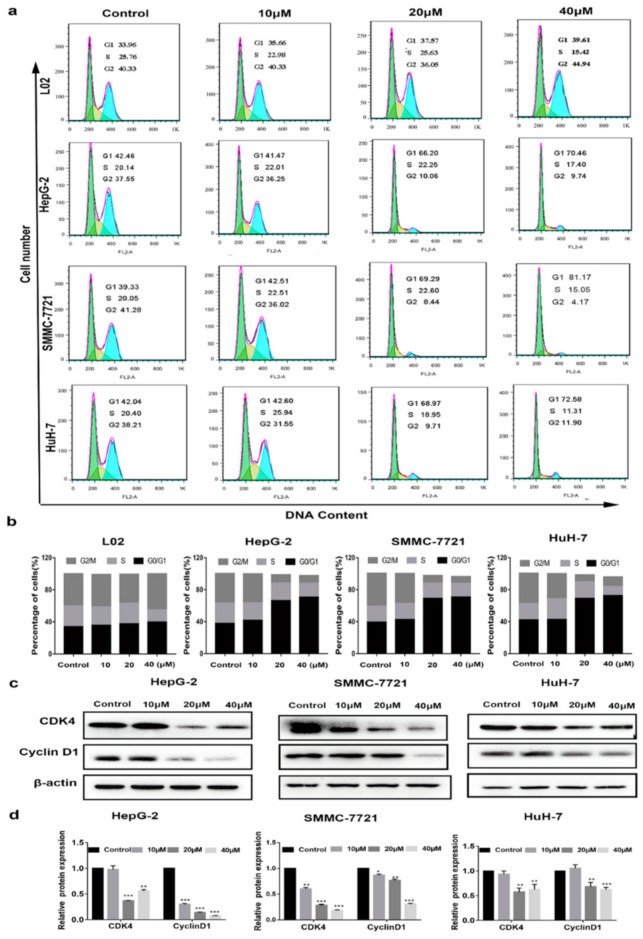
The cell cycle arrest in HCC cells and L02 incubated with compound **1**. (**a**,**b**) The cell cycle phases of the treated cells were evaluated by flow cytometry. The x-axis represents periodic distribution and the y-axis represents the number of cells. (**c**,**d**) The protein levels of cell cycle regulators, CDK4 and cyclinD1, were examined by Western blot analysis. * *p* < 0.05, ** *p* < 0.01, and *** *p* < 0.001 compared with the control.

Some anti-HCC drugs inhibit cell proliferation through cell cycle regulation, such as Ribociclib [15], Palbociclib [16] and Milciclib [17]. Cell cycle distribution was then examined by flow cytometry to determine how compound **1** affected HCC cell cycle progression. As illustrated in Figure 2a,b, the percentage of HCC cells in the G0/G1 phase increased significantly with the gradually increasing concentration of compound **1**, whereas the percentage of cells in the S and G2 phases decreased continually. Moreover, such a change was less pronounced in L02 cells as compared with the HCC cells. These data indicated that HCC cells arrested at the G0/G1 phase could be selectively induced by compound **1**. In addition, the evidence of a Western blot analysis showed that compound **1** downregulated the protein level of cyclin-dependent kinase 1 (CDK1) and cyclin D1 in a dose-dependent manner (Figure 2c,d). Therefore, compound **1** could induce HCC cell cycle arrest at the G0/G1 phase, and downregulate the expression of CDK1 and cyclin D1.

### 2.3. Selective Apoptosis Induced by Compound ***1***

**Figure 3:**
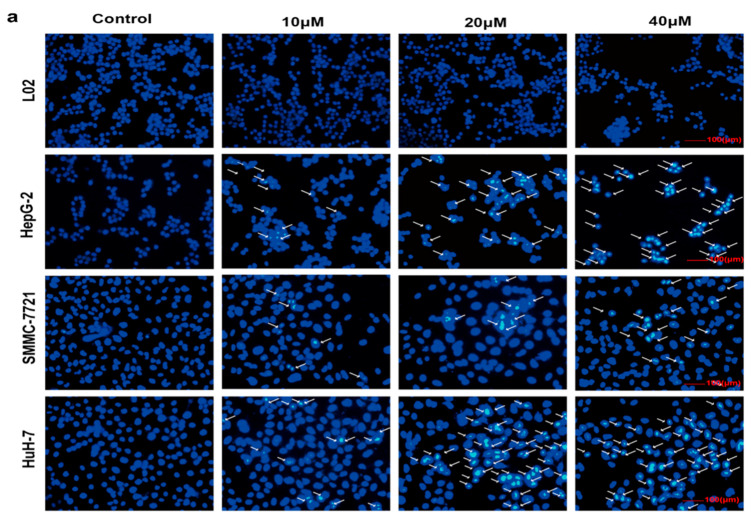

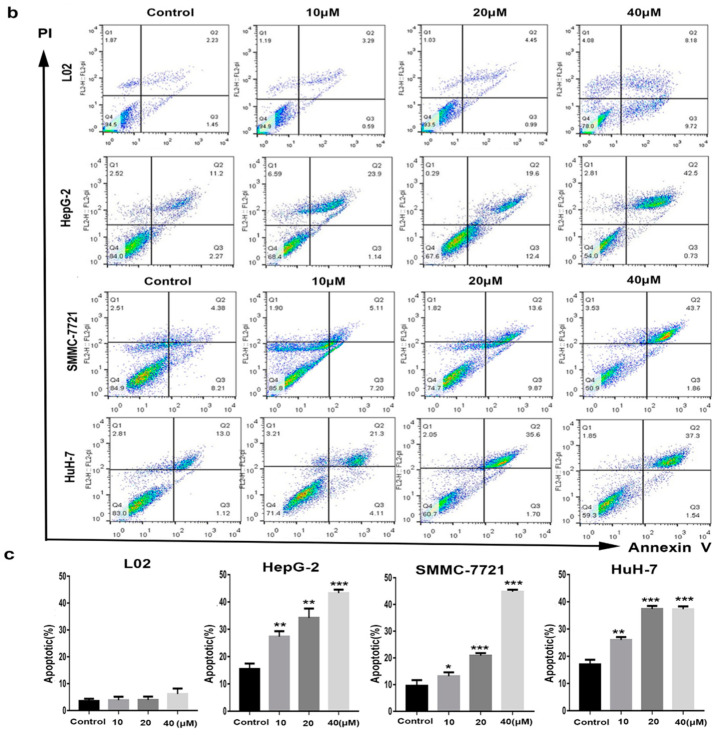
The apoptos is promoting effect of compound **1**. (**a**) The morphological changes in HCC cells and L02 cells. HCC cells and L02 cells were treated by compound **1** with various concentrations (0, 10, 20, and 40 μM) for 72 h. Apoptosis was evaluated by Hoechst 33342 dye to observe all morphological changes (bar = 100 μm). (**b**) HCC cells and L02 cells were treated by compound **1** with various concentrations (0, 10, 20 and 40 μM) for 72 h, and apoptosis effects were evaluated by PI/annexin V staining-based flow cytometry. (**c**) Representative histograms of the total apoptotic rate (% of total). The values are presented as mean ± SD (n = 3): * *p* < 0.05, ** *p* < 0.01, *** *p* < 0.001 compared to the control.

The induction of cancer cell apoptosis has been an efficient approach for most chemotherapeutic agents [18]. Apoptotic cells always present several typical morphology changes, including cell shrinkage, chromatin condensation, cytoplasm vacuolization, membrane blebbing, nuclear fragmentation, and the formation of apoptotic bodies [19]. To explore whether the proliferation inhibition of HCC cells induced by compound **1** was associated with cell apoptosis, various methods of apoptosis evaluation were applied in this study. Firstly, apoptosis was assessed by Hoechst staining. As shown in Figure 3a, HCC cells incubated with compound **1** displayed significant changes in cell morphology for 72 h when compared with the control group, such as chromatin condensation and fragmentation, indicating cell apoptosis. Nevertheless, these changes were not observed in L02, which reconfirmed the high selectivity of compound **1**. To further confirm whether compound **1** could induce apoptosis in cells, compound **1**-incubated HCC cells and L02 cells were stained with Annexin V and PI. As shown in Figure 3b,c, compared with the control group, the percentage of early and later apoptotic HepG-2 cells was significantly elevated from 13 to 43% after being treated by compound **1** (40 µM) for 72 h. The results of the SMMC-7721 cells and HuH-7 cells were similar to the HepG-2 cells. Surprisingly, there was no obvious pro-apoptotic effect on the L02 cells after being treated by compound **1** at the same concentration. Therefore, the apoptosis assay indicated that compound **1** induced selective apoptosis in HCC cells.

### 2.4. Mitochondrial Depolarization and ROS Generation Induced by Compound ***1***

**Figure 4:**
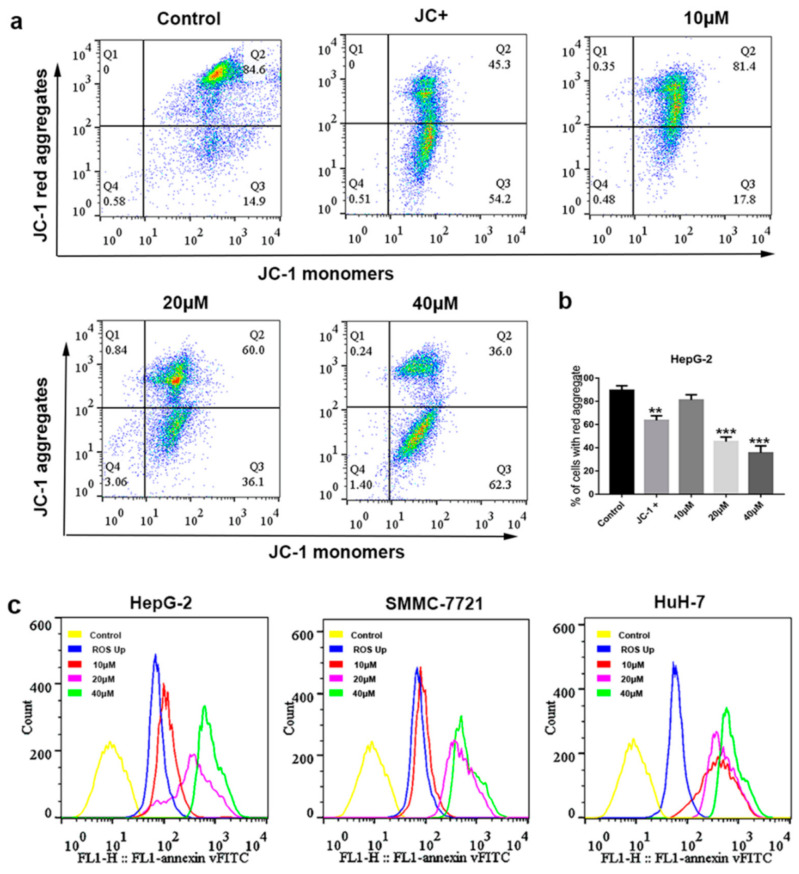
The loss of ΔΨm and ROS changes in HCC cells induced by compound **1**. (**a**) The change in ΔΨm in HepG-2 cells treated with various concentrations (0, 10, 20 and 40 μM) of compound **1** for 72 h were determined by flow cytometer analysis of JC-1. The regent JC+ from the JC-1 assay kit was used as a positive control. (**b**) Representative histograms of the loss of ΔΨm indicated by percentage (%) of cells with red aggregates. Data are represented as mean ± SD of three independent experiments. (**c**) HepG-2, SMMC-7721, and HuH-7 cells were first incubated with various concentrations (0, 10, 20 and 40 μM) of compound **1** for 72 h. Then, DCFH-DA (10 μM) was loaded, and the changes in ROS levels were analyzed by flow cytometry. ROSup was used as a positive control. The values are presented as mean ± SD (n = 3): ** *p* < 0.01, *** *p* < 0.001 compared to the control.

Mitochondria have been certified to play a core role in the initiation of apoptosis [20]. In particular, the release of diverse pro-apoptotic factors from mitochondria leading to ΔΨm change, which plays an important role in both extrinsic and intrinsic apoptosis [21]. In order to determine whether apoptosis induced by compound **1** was involved in the disruption of mitochondrial membrane integrity, a JC-1 fluorescent probe was used to test the change in ΔΨm. As shown in Figure 4a,b, the intensity of the red fluorescence was decreased in a dose-dependent manner after HepG-2 cells were treated with compound **1**. The loss of ΔΨm was observed by flow cytometry analysis. These changes in mitochondrial membrane were consistent with the data obtained above for the apoptosis assay.

The anti-HCC properties of a variety of chemotherapeutic drugs, such as salinomycin and oxaliplatin, have been ascribed to the generation of ROS [22,23]. Therefore, the effect of compound **1** on the ROS levels was investigated by the DCFH-DA stain method. The generation of ROS in HCC cells was detected after being treated with compound **1** in different concentrations (0, 10, 20 and 40 μM) for 72 h. ROSup, from the ROS assay kit, was used as the positive control. It was found that intracellular ROS generation significantly increased in a dose-dependent manner of compound **1** as compared to the control group (Figure 4c), which suggested that compound **1** could increase the level of ROS in HCC cells.

### 2.5. Mitochondrial Cytochrome c-Dependent Apoptosis Pathway Was Triggered by Compound ***1***

**Figure 5:**
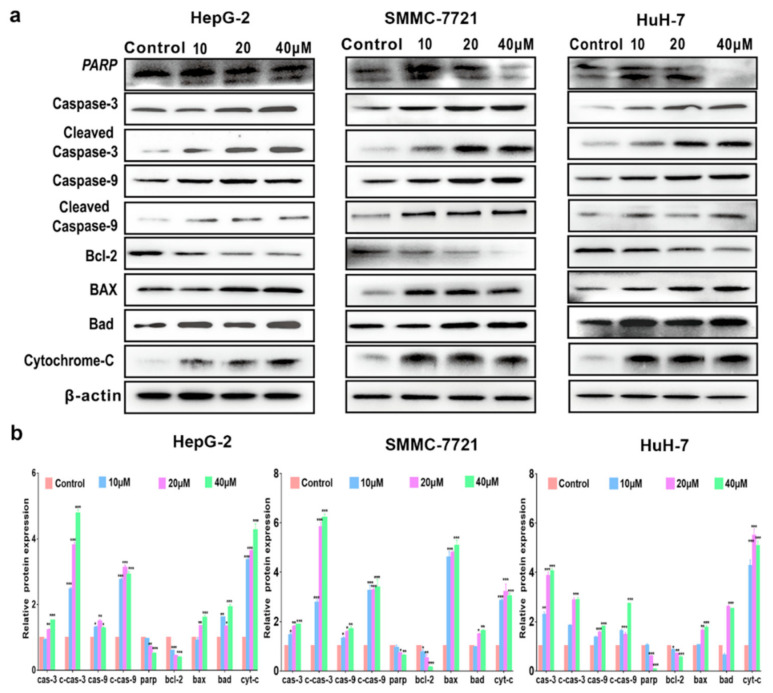
The apoptotic proteins analysis of HCC cells after being treated with compound **1**. (**a**) The expressions of Caspase-3, cleaved Caspase-3, Caspase-9, cleaved Caspase-9, Bcl-2, BAX, Bad, and Cytochrome *c* were determined by Western blot analysis. β-actin was used as internal control. (**b**) Representative histograms of the apoptotic proteins change. The density ratio of proteins to β-actin was shown as relative expression. Data are represented as mean ± SD of three independent experiments. * *p* < 0.05, ** *p* < 0.01, *** *p* < 0.001, the control compared with the cells treated with compound **1**.

To further clarify the underlying mechanism of compound **1**-induced apoptosis in HCC cells, a series of apoptosis-related proteins were detected in HCC cells by Western blot analysis. As shown in Figure 5a,b, the expression of pro-apoptotic proteins in HCC cells increased after being treated with compound **1** for 72 h, such as Cytochrome *c,* cleaved Caspase-9 and -3, cleaved PARP, BAX and Bad, while the expression of Bcl-2 significantly decreased.

**Figure 6:**
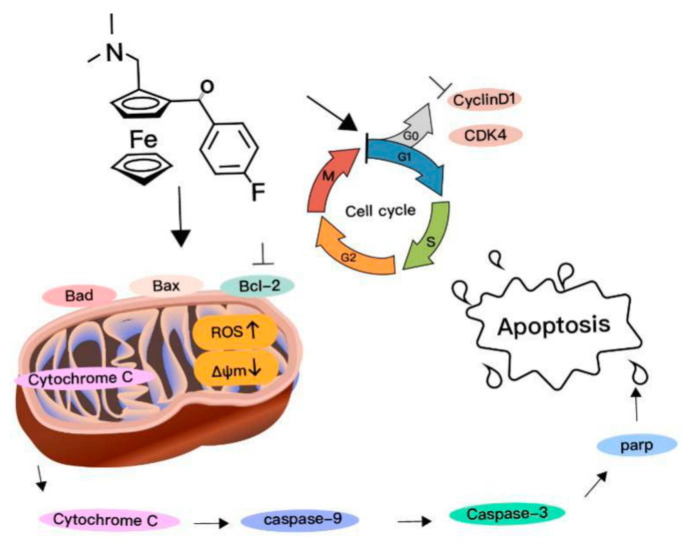
A proposed mitochondrial pathway for apoptosis in HCC cells induced by compound **1**.

The Bcl-2 family proteins (e.g., Bax, Bad and Bcl-2) control a critical step of apoptosis. With downregulation of Bcl-2, the Bad and Bax proteins act on the mitochondria to increase mitochondria permeability, resulting in the release of certain cellular components, such as Cytochrome *c* [24]. Moreover, the mitochondrial Cytochrome *c*, once released into the cytoplasm, was frequently involved in a chemically induced apoptotic signaling pathway and the activation of Caspase-9 and -3, leading to degradation of PARP [25]. Based on the results obtained above, a mitochondrial Cytochrome *c*-dependent death pathway was proposed for the apoptosis induced by compound **1** (Figure 6).

## 3. Discussion

A series of ferrocene derivatives were synthesized based on transition metal-catalyzed C−H functionalization [12]. In this work, it was clearly demonstrated that compound **1** showed a potent and selective effect on anti-HCC (Figure 1). Moreover, the cell cycle of HCC cells was blocked at G0/G1 after being treated by compound **1** for 72 h (Figure 2). Furthermore, compound **1** could induce apoptosis of HCC cells (Figure 3) via the mitochondrial pathway (Figure 6), i.e., the generation of ROS (Figure 4), the loss of ΔΨm (Figure 4)*,* the disequilibration of anti-apoptotic (Bcl-2) and pro-apoptotic (BAX, Bad) proteins, the release of mitochondrial Cytochrome *c,* and the activation of Caspase-9 and -3 (Figure 4 and Figure 5).

In the present study, it was clear that the ferrocene derivatives exhibited anti-HCC selectivity. The IC_50_ value of compound **1** against HepG-2 cells was more than 10-fold higher than that of L02 cells (Table S1). A significant degree of safety was presented when compared to the IC_50_ of sorafenib against L02 (0.35 μM). According to the structure–activity relationship analysis, it was clear that compounds **1** and **2**, with electron-withdrawing substituents at the benzoyl group, were more active. Compound **1** with para-substituent was also better than compound **2**. A further investigation on the relationship of structure–function is underway.

Cell cycle progression is the base of cell proliferation, tightly mediated by cell cycle-related proteins, such as cyclins and cyclin-dependent kinases (CDKs), and depends on the G1 checkpoint [26]. Cyclin D1 is a key regulator and leads to promotion of the G1 to S phase. Moreover, the binding of cyclin D1 with CDK 4/6 employs a key role in the G1-S checkpoint [27]. It has been reported that the overexpression of cyclin D1 is associated with cancers. Some antitumor agents have been proven to inhibit degradation of cyclin D1 [28,29]. In the present study, compound **1** could selectively induce G0/G1 arrest in HCC cell lines through downregulating cyclin D1 and CDK4. It was reported that capecitabine could induce arrest of the cell cycle in the G0/G1 phase to cause inhibition of colon cancer growth [30]. Metronomic capecitabine was developed for patients with HCC unresponsive to, or ineligible for, sorafenib treatment [31], and as second-line treatment of HCC after sorafenib failure [32]. Our study suggested that a combination treatment of HCC with compound **1** and capecitabine might achieve extra efficacy.

ROS has been identified to be involved in cell proliferation and apoptosis, as the production of ROS reduces the antioxidant capacity of cells and then elevates the sensitivity of cancer cells to chemotherapeutic agents [33]. HCC cells with high oxidative stress might be more vulnerable to damage with ROS generation [34]. A major source of cellular ROS is from mitochondria, which could stimulate mitochondrial dysfunction and thereby induce mitochondrial apoptosis to release Caspases family proteins [35]. The data obtained above showed that the accumulation of cellular ROS was enhanced in a dose-dependent manner in HCC cell lines (Figure 4c) after treatment with compound **1** (0–40 μM). Therefore, increasing levels of ROS might be partly involved in the apoptosis induced by compound **1**.

Activation of apoptosis can be classically triggered by two distinct pathways: the extrinsic, or death receptor, and the intrinsic, or mitochondrial. The loss of ΔΨm, a characteristic of mitochondrial injury, could induce the mitochondrial Cytochrome *c* to release into cytosol, which combines with Caspase to form a Caspase-activating complex [36]. Furthermore, signaling disruption of ΔΨm could reflect the alteration of mitochondrial membrane permeability and cause mitochondrial dysfunction—a pivotal and irreversible event in the apoptotic pathway [37]. Our findings displayed that compound **1** caused a loss of ΔΨm in HepG-2 cells (Figure 4a,b), implying that compound **1** might induce HCC cell apoptosis through the mitochondria-mediated pathway.

Previous studies demonstrated that the ratio of Bax/Bcl-2 impacts the ΔΨm, which, in turn, influences the initiation of apoptosis [38]. Bcl-2 family proteins include both pro-apoptotic proteins (e.g., BAX, Bad, and Bak) and anti-apoptotic proteins (e.g., Bcl-2, Bcl-w, and Bcl-xL). Disrupting the balance between anti-apoptotic Bcl-2 and pro-apoptotic Bax leads to apoptosis [39]. Therefore, the Bax/Bcl-2 ratio is an important indicator of apoptosis, which is the key regulator of mitochondrial pathway-mediated apoptosis [24]. Our studies showed that compound **1** could decrease the expression of anti-apoptotic Bcl-2 (Figure 5a,b) and increase the expression of pro-apoptotic BAX, while also increasing the Bax/Bcl-2 ratio in HCC cells. Therefore, this study suggested that compound **1** might trigger the mitochondrial apoptosis pathway by downregulating the expression of Bcl-2 and upregulating the expression of BAX/Bad.

Meanwhile, the mitochondrial-mediated intrinsic apoptotic pathway may involve Cytochrome *c* release and Caspase-9 activation [40]. Caspase-9 is a major initiator protein of Caspase cascade reaction in the mitochondrial apoptosis pathway, which could activate the final executor of apoptosis, such as Caspase-3 [41]. Previous investigations showed that Caspase-9 and Caspase-3 could be activated once Cytochrome *c* was released from the inner mitochondrial membrane to the cytosol, leading to the cleavage of PARP and the final apoptosis of a cell [42]. To understand the molecular mechanism underlying apoptosis caused by compound **1**, we focused on the potential involvement of the Caspase cascade in the process of apoptosis. The increase in Caspase-9, -3, and PARP cleavage in HCC cells demonstrated that apoptosis was induced by compound **1** (Figure 5a,b).

In summary, compound **1** exhibited significant antitumor efficacy and good selectivity in vitro, and HCC cell death induced by compound **1** was proposed to be involved in the relative mechanism (Figure 6). Compound **1** may therefore have the potential to be further developed as a promising anti-HCC agent.

## 4. Materials and Methods

### 4.1. Materials

Compounds **1**, **2**, and **3** were synthesized according to the procedure developed by our group [12]. They were dissolved in dimethyl sulfoxide (DMSO), the concentration of which never exceeded 0.1% (*v*/*v*); 50 mM of stock solution was stored at −20 °C. The Cell counting Kit-8 (CCK-8), Hoechst 33258, JC-1, and the ROS assay kit were purchased from Beyotime Institute of Biotechnology Company (Shanghai, China). The annexin V-FITC and propidium iodide (PI) kit was obtained from BD Pharmingen (BD, San Diego, CA, USA). The primary antibodies used are as follows: CDK4 (D9G3E, Cell Signaling Technology, Danvers, MA, USA), Cyclin D1 (E3P5S, Cell Signaling Technology), Caspase-3 and cleaved Caspase-3 (D3R6Y, Cell Signaling Technology), PARP (46D11, Cell Signaling Technology), Cytochrome *c* (AF0146, Affinity, Changzhou, China), Caspase-9 (AF6348, Affinity), cleaved Caspase-9 (D8I9E, Cell Signaling Technology), BAX (D2E11, Cell Signaling Technology), Bcl-2 (D17C4, Cell Signaling Technology), Bad (D24A9, Cell Signaling Technology), and β-actin (AF0003, Beyotime Biotechnology, Shanghai, China). All other chemicals used are commercially available and reagent grade.

### 4.2. Cells Culture

Human hepatoblastoma cells (HepG2, SMMC-7721, and HuH-7) and normal human cell line (L02) were obtained from the Cell Bank of Type Culture Collection of the Chinese Academy of Sciences (Shanghai, China). SMMC-7721 cells, HepG-2 cells, and HuH-7 cells were cultured in DMEM (Gibco, Waltham, MA, USA), supplemented with 10% FBS (Gibco), penicillin (100 U/mL), and streptomycin (100 U/mL). L02 cells were cultured in RPMI1640 (Gibco), supplemented with 10% FBS, penicillin (100 U/mL) and streptomycin (100 U/mL). All cells were incubated at 37 °C with 5% CO_2_.

### 4.3. CCK-8 Assay

Cells were seeded in 96-well plates at a density of 5 × 10^3^ cells/well, and incubated for 12 h prior to treatment. Compounds were added in 0, 10, 20, 40, 80 and 120 μM for each cell population. The 0 μM group was set as the control group. Cell viability was measured after 24, 48 and 72 h, respectively. The CCK-8 (10 μL) was added to each well and incubated for 2 h in the dark at 37 °C. The absorbance of the solution was measured at 450 nm using enzyme labeling. The data were expressed as inhibited cell growth 50% (IC_50_), using Graph Prism 7 to determine IC_50_ values. All of the experiments were repeated triply.

### 4.4. Cell Cycle Analysis

HepG-2, SMMC-7721, HuH-7, and L02 cells were treated with a gradually increasing concentration of compound **1** (0, 10, 20 and 40 μM) for 72 h. Cells were collected, then washed by cold phosphate-buffered saline (PBS) and fixed in 75% ethanol overnight at 4 °C. Cells were centrifuged and incubated with RNase (Sigma, St. Louis, MO, USA) at 37 °C for 30 min before being stained with PI. Cells were then resuspended in 500 μL of 1X binding buffer after the residual RNase was removed. The DNA contents and cell cycle distribution were analyzed with flow cytometry (FACS Calibur, BD).

### 4.5. Apoptosis Analysis

The cells (HepG-2, SMMC-7721, HuH-7 and L02) were incubated in a 6-well plate and treated by compound **1** (0, 10, 20 and 40 μM) for 72 h. The cells were fixed for 10 min after the culture medium was removed and 0.5 mL of fixative was added. Cells were washed with PBS. Then, the morphological changes of apoptotic cell nuclei were detected with a fluorescence microscope.

HepG-2, SMMC-7721, HuH-7, and L02 cells were treated with compound **1** of various concentrations (0, 10, 20 and 40 μM) for 72 h. Then, cells were collected and washed 3 times with cold PBS buffer. Cells were centrifuged and then suspended in 500 μL of 1X binding buffer. Finally, the cells were stained with Annexin V-FITC (5 μL) and PI (5 μL) at 37 °C for 15 min in the dark, and then examined by flow cytometry. The percentages of apoptotic cells were determined by flow cytometry on Flow Jo software.

### 4.6. ROS Detection

Intracellular ROS production was detected using the cell-permeable fluorogenic probe 2′,7′-dichlorodihydrofluorescein diacetate (DCFH-DA). The cells were incubated with 10 mM DCFH-DA at 37 °C for 15 min. Following this, the cells were treated with compound **1** (0, 10, 20 and 40 μM) for 72 h, and then examined by flow cytometry.

### 4.7. The ΔΨm Analysis 

HepG-2 cells were plated in a 12-well plate, then treated with compound **1** (0, 10, 20 and 40 μM) for 72 h. The ΔΨm of HepG-2 cells was analyzed by the JC-1 staining method after HepG-2 cells were collected, centrifuged, and then resuspended in 0.5 mL of cell culture medium (0.5–1 × 10^6^ cells/mL). The JC-1 dyeing working solution was added to cells with culture medium, which was incubated at 37 °C for 20 min. Cells were washed with JC-1 staining buffer (1X) 3 times. Then, cells were resuspended with JC-1 staining buffer (1X) and examined with a flow cytometer and analyzed with Flow Jo software: excitation wavelength Ex = 488 nm and emission wavelength FL1 (Em = 579 nm); FL2 (Em = 599 nm).

### 4.8. Western Blot Analysis

HepG-2, SMMC-7721 and HuH-7 cells (1 × 10^6^ cells/dish) were seeded in a 6-well plate. After 12 h, the cells were treated by compound **1** with different concentrations (0, 10, 20 and 40 μM) for 72 h. Cells were then collected, washed by PBS, lysed in radioimmunoprecipitation assay (RIPA) buffer (Beyotime Biotechnology), and supplemented with a protease inhibitor cocktail (Thermo Scientific, Waltham, MA, USA) at 4 °C for 30 min for total protein extracts. Lysates were removed by centrifugation at 15,000 g for 20 min at 4 °C. The protein extracts were separated using 10% SDS–polyacrylamide gel electrophoresis, and then transferred to nitrocellulose membrane (Thermo Scientific). The membranes were blocked in 5% skimmed milk (BD) at room temperature for 3 h, and then incubated with primary antibodies overnight at 4 °C. Next, the membranes were washed 3 times with 5% Tween–phosphate-buffered saline (PBS) and incubated with appropriate horseradish peroxidase (HRP) conjugated secondary antibodies for 1 h at room temperature. The protein bands were visualized with BeyoECL Star (Beyotime Biotechnology) after cleaning the membrane 3 times with 5% Tween–PBS.

### 4.9. Statistical Analysis

Statistical significance was assessed by Student’s *t*-test or analysis of variance (ANOVA) using GraphPad Prism 7 (GraphPad software).

## Data Availability

Not applicable.

## Supplementary Materials

The following are available online at https://www.mdpi.com/1422-0067/22/6/3097/s1, Figure S1 shows chemical structures of compounds **1**, **2**, and **3**. The IC_50_ (μM) of compounds **1**, **2**, **3** and sorafenib against HCC and L02 cell lines for 72 h were available at Table S1.

## Abbreviations

HCC	Human hepatocellular carcinoma
ROS	Reactive oxygen species
FBS	Fetal bovine serum
DMSO	Dimethyl sulfoxide
PBS	Phosphate-buffered saline
RIPA	Radio-immunoprecipitation assay
ΔΨm (MMP)	Mitochondrial membrane potential
DCFH-DA	Dichlorodihydrofluorescein diacetate
CDKs	Cyclin-dependent kinases
