# Supplementary material for: Novel Ferrocene Derivatives Induce G0/G1 Cell Cycle Arrest and Apoptosis through the Mitochondrial Pathway in Human Hepatocellular Carcinoma
Source: Int J Mol Sci. 2021 Mar 18;22(6):3097. doi: 10.3390/ijms22063097 (PMC8003055; doi:10.3390/ijms22063097)
Supplement: Supplementary file 1 [file ijms-22-03097-s001.pdf]

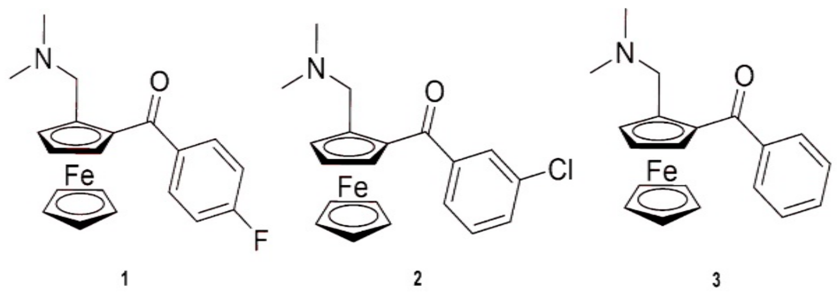

Figure S1. Chemical structures of compounds 1, 2 and 3.

Table S1 the IC_{50} (μM) of sorafenib, compounds 1, 2 and 3 against HCC and L02 cell lines

	L02	HepG-2	SMMC-7721	HuH-7
Sorafenib	0.35±2.11	0.41±1.20	0.94±0.55	0.64±3.21
1	209.90±1.80	18.80±5.42	25.62±0.90	20.31±3.31
2	187.52±2.84	22.42±4.60	35.59±3.02	34.40±0.70
3	168.70±0.18	20.00±1.36	38.07±1.25	19.22±1.60

The cells viability results of 72h are expressed as the mean ± standard (SD), n=3.
